# Meta-analysis of gene expression profiling reveals novel basal gene signatures in MCF-10A cells transformed with cadmium

**DOI:** 10.18632/oncotarget.27734

**Published:** 2020-09-29

**Authors:** Katrina Blommel, Carley S. Knudsen, Kyle Wegner, Swojani Shrestha, Sandeep K. Singhal, Aaron A. Mehus, Scott H. Garrett, Sonalika Singhal, Xudong Zhou, Brent Voels, Donald A. Sens, Seema Somji

**Affiliations:** ^1^Department of Pathology, University of North Dakota, School of Medicine and Health Sciences, Grand Forks, ND 58202, USA; ^2^Department of Science, Cankdeska Cikana Community College, Fort Totten, ND 58335, USA; ^*^These authors contributed equally to this work

**Keywords:** breast cancer, meta-analysis, cadmium, MCF-10A, basal subtype

## Abstract

Cadmium (Cd^2+^) is an environmental toxicant and a human carcinogen. Several studies show an association of Cd^2+^ exposure to the development of breast cancer. Previously, we have transformed the immortalized non-tumorigenic cell line MCF-10A with Cd^2+^ and have demonstrated that the transformed cells have anchorage independent growth. In a separate study, we showed that transformation of the immortalized urothelial cells with the environmental carcinogen arsenite (As^3+^) results in an increase in expression of genes associated with the basal subtype of bladder cancer. In this study, we determined if transformation of the MCF-10A cells with Cd^2+^ would have a similar effect on the expression of basal genes. The results of our study indicate that there is a decrease in expression of genes associated with keratinization and cornification and this gene signature includes the genes associated with the basal subtype of breast cancer. An analysis of human breast cancer databases indicates an increased expression of this gene signature is associated with a positive correlation to patient survival whereas a reduced expression/absence of this gene signature is associated with poor patient survival. Thus, our study suggests that transformation of the MCF-10A cells with Cd^2+^ produces a decreased basal gene expression profile that correlates to patient outcome.

## INTRODUCTION

The relationship between exposure to cadmium (Cd^2+^) and the role it might play in the development and progression of breast cancer is controversial. There are case-controlled studies, which show that higher urinary levels of Cd^2+^ correlate positively with a significant increase in the risk of breast cancer [1, 2]. Several meta-analyses have also shown a link between the urinary level of Cd^2+^ and the risk of breast cancer [3, 4]. However, two prospective studies have concluded that there is no association between urinary Cd^2+^ and the risk of breast cancer [5, 6]. Although, most studies on Cd^2+^ intake in human subjects do not support a link with the risk of breast cancer [7, 8], a recent study does associate dietary Cd^2+^ intake with an increased risk for breast cancer [9]. Another recent study suggests that individuals having chronic long-term exposure to Cd^2+^ via air pollution, while showing no overall breast cancer risk, show evidence for a decreased risk for having estrogen receptor negative (ER^-^) and ER^-^/ progesterone receptor negative (PR^-^) breast cancers [10]. There is also evidence that Cd^2+^ has estrogenic activity, can interact with the ER in cell culture studies and can mimic estrogen effects in the uterus and mammary gland of animals [11–13]. Recent studies using MCF-7 cells also suggests that Cd^2+^ exposure can decrease the dependence of cells on ERα [14]. These studies provide evidence that Cd^2+^ could have a yet undefined role in the development and progression of breast cancer.

This laboratory has previously transformed MCF-10A cells by exposure to 1 μM Cd^2+^ [15]. The Cd^2+^-transformed cells form colonies in soft agar but are not tumorigenic and do not form tumors when injected subcutaneously or into the peritoneal cavity of immune compromised mice. The MCF-10A cell line is frequently used as a cell culture model of “normal” breast epithelial cells that have undergone spontaneous immortalization following isolation from benign proliferative breast tissue [16]. This cell line possesses a basal-like gene expression pattern, is ER^-^ and PR^-^, and shows no evidence of invasiveness or tumor formation in immune compromised mice [16–18]. The basal-like gene signature of the MCF-10A cells and the ability to undergo transformation with Cd^2+^ is of interest since this laboratory has previously shown that urothelial cells malignantly transformed with Cd^2+^ or As^3+^ express a basal gene signature in cell culture and in tumor heterotransplants [19, 20]. This basal gene signature correlates with the development of muscle-invasive disease and poor outcomes in patients with urothelial cancer [21–23]. The basal gene signature associated with muscle-invasive bladder cancer (MIBC) consists of 11 induced genes (CD44, CDH3, TP63, KRT1, KRT5, KRT6A, KRT6B, KRT6C, KRT 14, KRT16, and KRT 17).

The identification of the basal gene signature for MIBC allowed this laboratory to re-examine basal gene expression from an older Affymetrix™ U133 Plus 2.0 global gene expression profile obtained for MCF-10A cells and their Cd^2+^-transformed counterpart. Thus, the first goal of this study was to validate a global gene analysis study that suggested an alteration in the basal gene expression in MCF-10A cells transformed with Cd^2+^ and to determine if these basal genes would implicate other cellular pathways. In addition, we also determined if the gene signatures associated with basal gene expression might correlate to outcomes for patients with breast cancer.

## RESULTS

### Cadmium transformation of MCF-10A cells decreases the expression of basal genes

**Figure 1:**
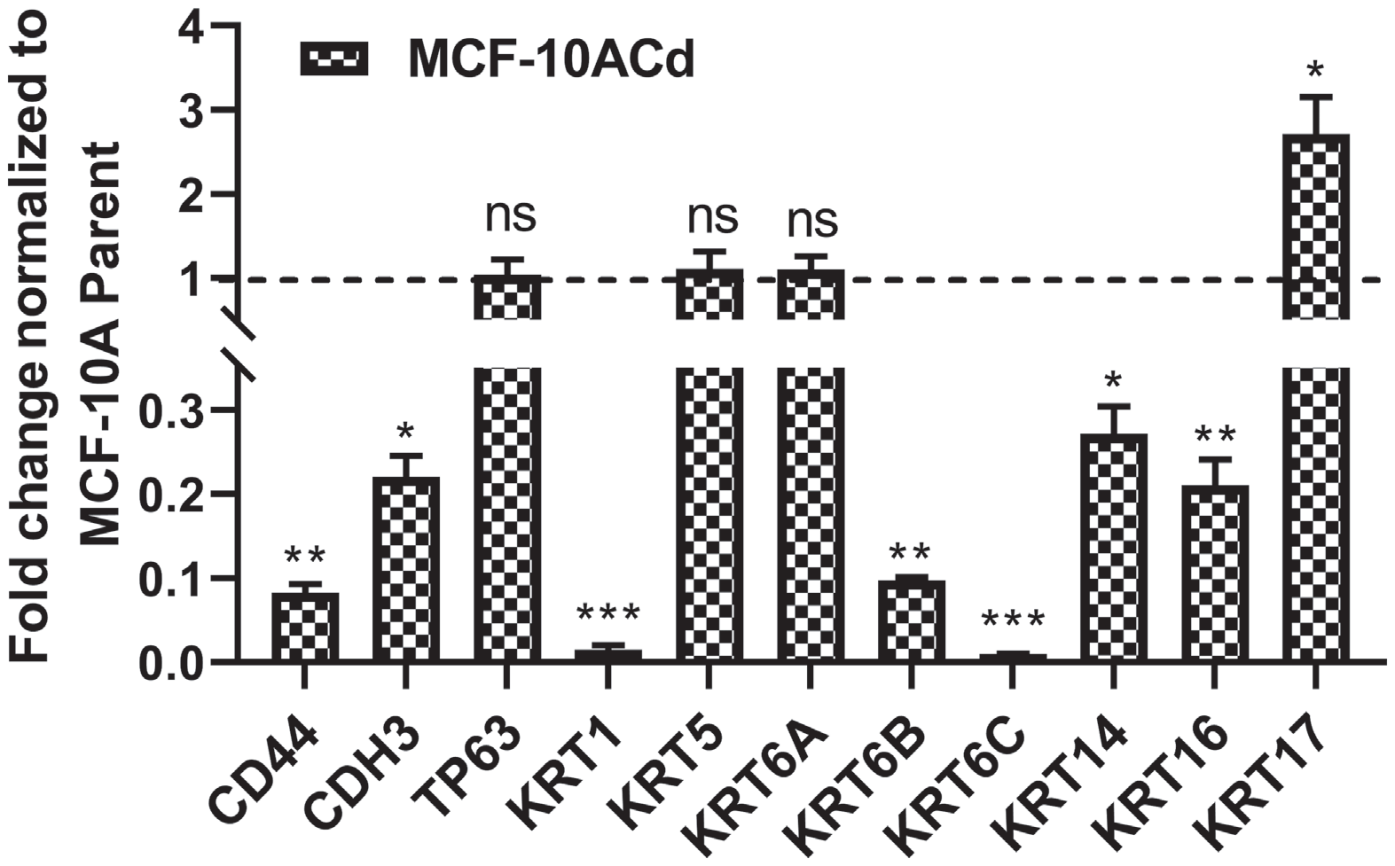
Expression of basal markers in MCF10A cells transformed with Cd^2+^. Real time RT-PCR analysis of CD44, CDH3, TP63, KRT1, KRT5, KRT6A, KRT6B, KRT6C, KRT14, KRT16, and KRT17 in MCF-10A parent and MCF-10ACd cells. The expression level of each gene in the MCF-10ACd cells is normalized to the MCF-10A parent cells and plotted as fold change. The dotted line represents normalized value of gene expression in MCF-10A parent cells. ^***^; ^**^; ^*^Indicates significant difference in gene expression level compared to the MCF-10A parent cells at *p*-value of ≤ 0.001; ≤ 0.01; ≤ 0.05, respectively; ns represents change in expression is not significant.

In a previous study, we have shown that the basal gene signature associated with MIBC was highly expressed in a cell culture model of As^3+^-induced bladder cancer [19]. Microarray analysis of differentially expressed genes between the parent MCF-10A cells and the MCF-10A cells transformed with Cd^2+^ (MCF-10ACd) showed that out of these 11 genes, eight of them (CD44, KRT1, KRT5, KRT6A, KRT6B, KRT14, KRT16, and KRT17) had decreased expression in the MCF-10ACd cells. There was no differential expression of three of the genes CDH3, TP63 and KRT6C in the array. The expression of the 11 MIBC associated genes was confirmed by real-time RT-PCR in the MCF-10 and MCF-10ACd cells and the data is presented in Figure 1. These results confirmed the decreased expression of CD44, KRT1, KRT6B, KRT14 and KRT16. In addition, there was also a decrease in expression of CDH3 and KRT6C in the MCF-10ACd cells. There was no change in the expression levels of TP63, KRT5, KRT6A, whereas the expression of KRT17 was increased in the MCF-10ACd cells. Thus, our data shows that the majority of the genes associated with a basal gene profile have decreased expression in the MCF-10ACd cells. Many of these basal genes are also associated with the potential stem/progenitor basal cells [21].

### Keratinization gene signature of MCF-10ACd cells

An analysis of the genes repressed in the MCF-10ACd cells was performed using the Reactome Pathway Knowledge Base [24] and it was determined that 29 genes associated with keratinization and formation of the cornified envelope were repressed in the MCF-10ACd cells when compared to the MCF-10A parent cells. These 29 genes are listed in Supplementary Table 1. The basal genes repressed in the MCF-10ACd cells (Figure 1) are a part of a larger group of genes involved in keratinization and cornification and this is in contrast to the UROtsa cells transformed with As^3+^ that have an increased expression of these 29 genes [20]. In MIBC patients, an increase in basal gene expression correlates with increase aggressiveness of the disease [21].

### Keratinization gene signature and breast cancer survival

The 29 genes identified by the Reactome Pathway Knowledge Base for MCF-10ACd cells were each analyzed separately and as a group to determine if any of them were linked with survival in patients with breast cancer. The 29 genes named as the Basal Keratinization Cluster (BKC) are shown in Table 1. An independent KM-plotter survival analysis of each gene identified 14 of the genes from BKC that are significant in their ability to separate good verses poor survival based upon median cutoff of low vs high gene expression value of all breast cancer populations. These 14 genes are: PKP3, DSG3, DSC2, DSC3, KLK5, PERP, PPL, KRT10, KRT1, SPRR3, SPTAN1, CTSA, DSC1, and JUP and this group is designated as the Basal Keratinization Cluster Sig (BKCsig). In addition, six genes (KRT6B, EBS4, KRT17, KRT5, DSG3, PPL) were found to be significant within the ER^+^ patients only with *p* value threshold 0.01 i.e., *p*-value < 0.01. No single gene was significant in the ER^-^ population. The direction of survival curves were mixed as high expression of some genes was a significant predictor of poor survival; whereas, it was the opposite in a few other cases (Table 1).

**Table 1:** Survival analysis of selective genes with hazard ratio (HR), 95% confidence interval (CI) and P-value

Affymetrix_ID	Gene	All Cancers				ER^+^ Cancers				ER^−^ Cancers			
		HR^*^	Low^**^	High^***^	*P*-value	HR^*^	Low^**^	High^***^	*P*-value	HR^*^	Low^**^	High^***^	*P*-value
203691_at	PI3	1.12	1.01	1.25	3.09E-02	1.05	0.89	1.23	5.80E-01	0.80	0.64	1.00	5.30E-02
214580_x_at	KRT6A	0.97	0.87	1.08	6.00E-01	0.98	0.83	1.15	7.90E-01	1.13	0.90	0.41	2.90E-01
200606_at	DSP	1.04	0.93	1.16	4.90E-01	0.98	0.83	1.16	8.30E-01	1.03	0.82	1.29	7.80E-01
206008_at	TGM1	0.91	0.82	1.02	1.10E-01	1.02	0.86	1.20	8.40E-01	0.92	0.74	1.16	5.00E-01
1553973_a_at	SPINK6	0.88	0.76	1.03	1.10E-01	0.70	0.52	0.94	1.80E-02	0.91	0.66	1.27	5.90E-01
213680_at	KRT6B	0.95	0.85	1.06	3.50E-01	0.80	0.68	0.94	6.90E-03	0.94	0.75	1.18	6.00E-01
209125_at	KRT6C	0.97	0.87	1.08	5.90E-01	1.06	0.90	1.25	4.70E-01	1.15	0.92	1.44	2.20E-01
213796_at	SPRR1A	1.01	0.9	1.12	8.80E-01	1.07	0.91	1.26	4.00E-01	1.03	0.83	1.30	7.70E-01
205064_at	SPRR1B	0.81	0.73	0.90	1.60e-04	0.99	0.84	1.16	8.70E-01	0.87	0.69	1.09	2.20E-01
209351_at	KRT14	0.79	0.71	0.88	1.90E-05	0.66	0.56	0.77	4.30E-07	1.06	0.84	1.32	6.30E-01
209800_at	KRT16	1.24	1.11	1.38	1.10E-04	0.91	0.77	1.07	2.70E-01	1.19	0.95	1.49	1.30E-01
200752_s_at	CANPL1	0.94	0.84	1.04	2.40E-01	0.95	0.81	1.12	5.40E-01	0.90	0.71	1.12	3.40E-01
205157_s_at	KRT17	0.94	0.84	1.05	2.80E-01	0.72	0.61	0.85	8.20E-05	0.99	0.79	1.24	9.30E-01
201015_s_at	JUP	1.07	0.96	1.19	2.50E-01	1.05	0.89	1.23	6.00E-01	1.12	0.96	1.51	1.10E-01
207023_x_at	KRT10	0.96	0.86	1.07	4.80E-01	1.13	0.96	1.33	1.60E-01	0.95	0.76	1.20	6.90E-01
207935_s_at	KRT13	0.8	0.72	0.89	5.90E-05	0.88	0.75	1.04	1.40E-01	0.95	0.76	1.19	6.50E-01
205900_at	KRT1	0.76	0.69	0.85	1.40E-06	1.06	0.90	1.25	5.00E-01	0.90	0.72	1.13	3.60E-01
232082_x_at	SPRR3	1.01	0.87	1.18	8.80E01	0.94	0.71	1.26	7.00E01	1.09	0.78	1.52	6.10E-01
201820_at	KRT5	0.84	0.75	0.94	1.60E-03	0.73	0.62	0.87	2.20E-04	0.91	0.73	1.14	4.20E-01
209873_s_at	PKP3	0.92	0.82	1.02	1.20E-01	1.10	0.94	1.30	2.40E-01	0.88	0.70	1.10	2.70E-01
235075_at	DSG3	0.82	0.71	0.96	1.40E-02	0.60	0.45	0.81	6.60E-04	1.16	0.83	1.61	3.80E-01
215235_at	SPTAN1	0.63	0.57	0.71	2.20E-16	0.81	0.69	0.95	1.10E-02	1.09	0.87	1.37	4.40E-01
204971_at	CSTA	1.17	1.05	1.31	4.10E-03	0.96	0.82	1.13	6.30E-01	1.10	0.88	1.38	4.00E-01
207324_s_at	DSC1	0.91	0.81	1.01	8.40E02	0.93	0.79	1.09	3.70E-01	1.11	0.89	1.39	3.70E-01
226817_at	DSC2	1.34	1.15	1.57	2.00E04	1.00	0.75	1.33	9.90E-01	0.80	0.57	1.11	1.90E-01
244107_at	DSC3	0.82	0.70	0.96	1.10E-02	0.80	0.60	1.07	1.40E-01	0.76	0.55	1.06	1.10E-01
222242_s_at	KLK5	0.86	0.77	0.96	7.40E-03	0.88	0.75	1.04	1.40E-01	0.82	0.65	1.02	8.00E-01
236009_at	PERP	1.14	0.98	1.33	9.80E-02	1.04	0.78	1.38	8.10E-01	0.86	0.62	1.20	3.70E-01
203407_at	PPL	0.77	0.69	0.86	3.50E-06	0.68	0.57	0.80	2.90E-06	1.11	0.89	1.39	3.60E-01

* HR: Hazard Ratio, ** Low: 95% Confidence Interval, *** High: 95% Confidence Interval.

### Association of each independent gene to the various breast cancer subtypes

**Figure 2:**
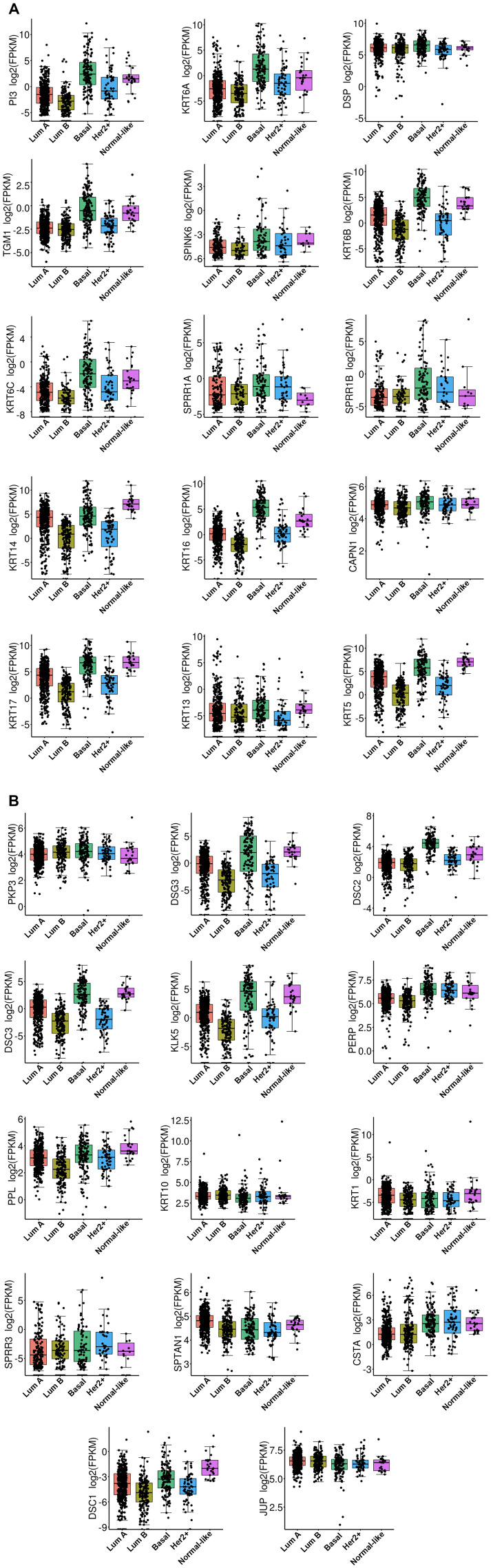
The box plot of RNA expression on TCGA data across BC subtype for all 29 gene. (**A**) RNA expression of genes PI3, KRT6A, DSP, TGM1, SPINK6, KRT6B, KRT6C, SPRR1A, SPRR1B, KRT14, KRT16, CAPN1, KRT17, KRT13, and KRT5. (**B**) RNA expression of genes PKP3, DSG3, DSC2, DSC3, KLK5, PERP, PPL, KRT10, KRT1, SPRR3, SPTAN1, CSTA, DSC1, and JUP. X-axis represent the BC subtype classified by PAM50 algorithm and named as LumA (Luminal A), LumB (Luminal B), Basal (Triple negative BC), Her2 (HER2+), and Normal-like. The y-axis is corresponding to log2 Fragments Per Kilobase of transcript per Million mapped reads (FPKM) value of RNA-Seq data. The corresponding gene name is provided on y-axis.

**Figure 3:**
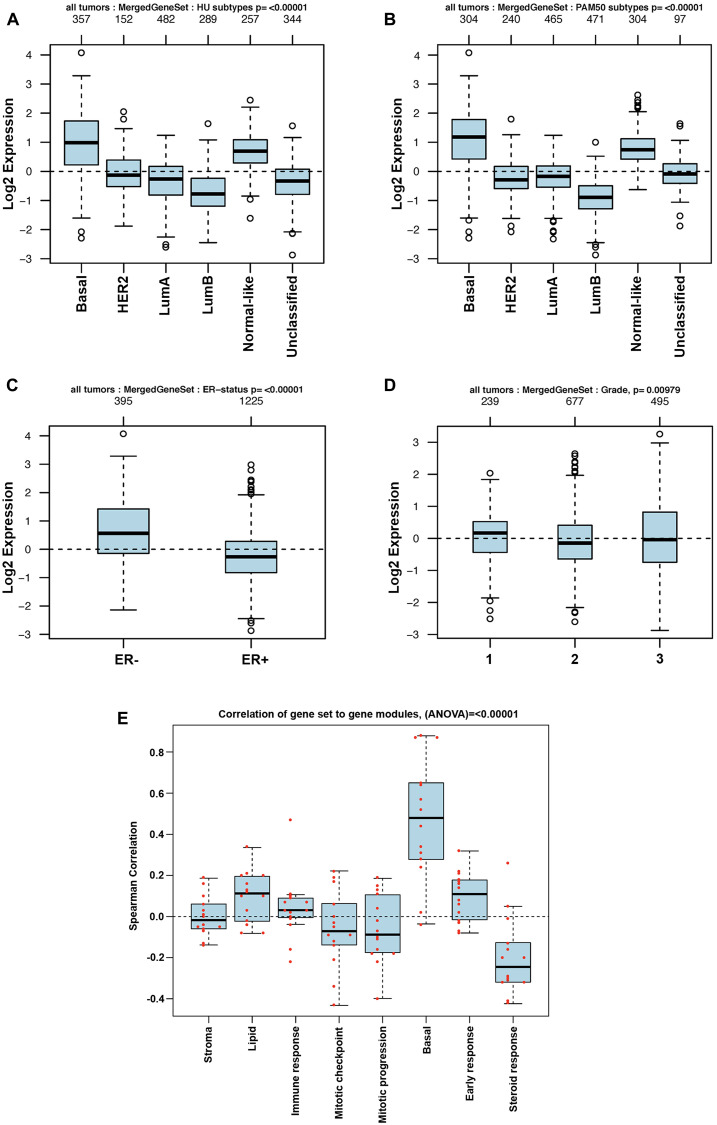
GOBO analysis of BKC gene expression signature. Box plot for BKC gene expression signatures for tumor samples stratified according to (**A**) HU gene expression subtype, (**B**) PAM50 gene expression subtype, (**C**) ER status, and (**D**) histological grade. (**E**) Pair-wise Spearman correlation of genes in the BKC signature to each gene in the eight functionally know gene signature to understand the functional relevance of our new gene signatures in breast cancer progression. Red dots indicate actual correlation values.

**Figure 4:**
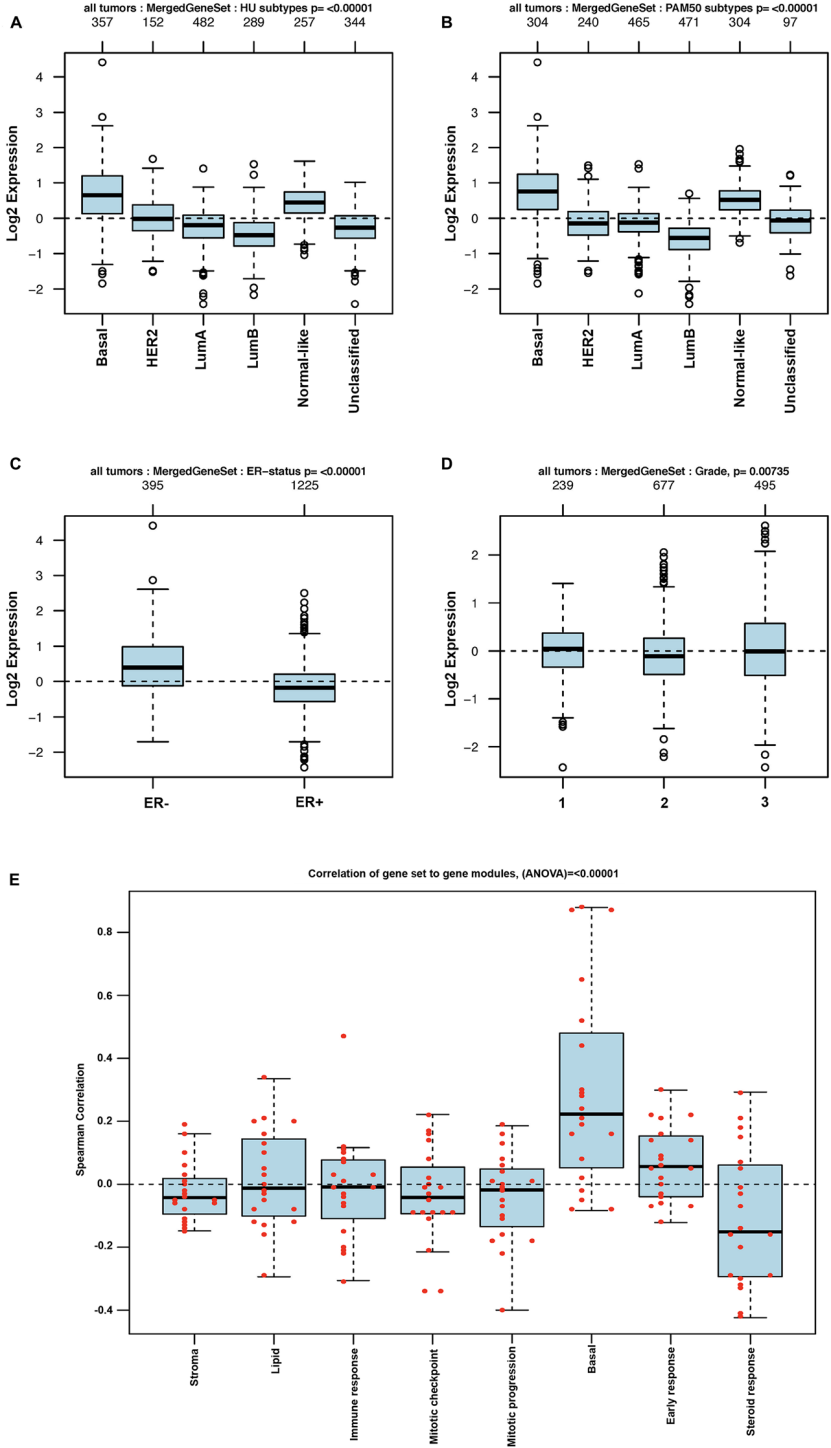
GOBO analysis of BKCsig gene expression signature. Box plot for BKCsig expression signatures for tumor samples stratified according to (**A**) HU gene expression subtype, (**B**) PAM50 gene expression subtype, (**C**) ER status, and (**D**) histological grade. (**E**) Pair-wise Spearman correlation of genes in the BKCsig signature to each gene in the eight functionally know gene signature to understand the functional relevance of our new gene signatures in breast cancer progression. Red dots indicate actual correlation values.

The TCGA dataset containing 1,102 breast cancer samples was used to define the association of each of the 29 genes in the BKC group with the breast cancer subtypes (Luminal A, Luminal B, Basal [Triple negative breast cancer], HER2+ and the Normal-like). The results of this analysis are shown in Figure 2. The results show that 21 out of the 29 genes in the BKC group are mainly enriched in the basal subtype of breast cancer. These results are further supported using the GOBO breast cancer patient population (Figures 3 and 4). These results show that the BKC (Figure 3) and BKCsig (Figure 4) gene signatures are elevated in basal breast cancers in the HU subtype (Figures 3A and 4A) and the PAM50 expression subtype (Figures 3B and 4B). However, there is no association with the ER status (Figures 3C and 4C) or histological grade of the tumor (Figures 3D and 4D). When compared to each of the eight functionally known gene expression modules, there is a positive correlation with the lipid module and the early response (proliferation) module (Figures 3E and 4E).

### Survival analysis of each of the gene signatures

**Figure 5:**
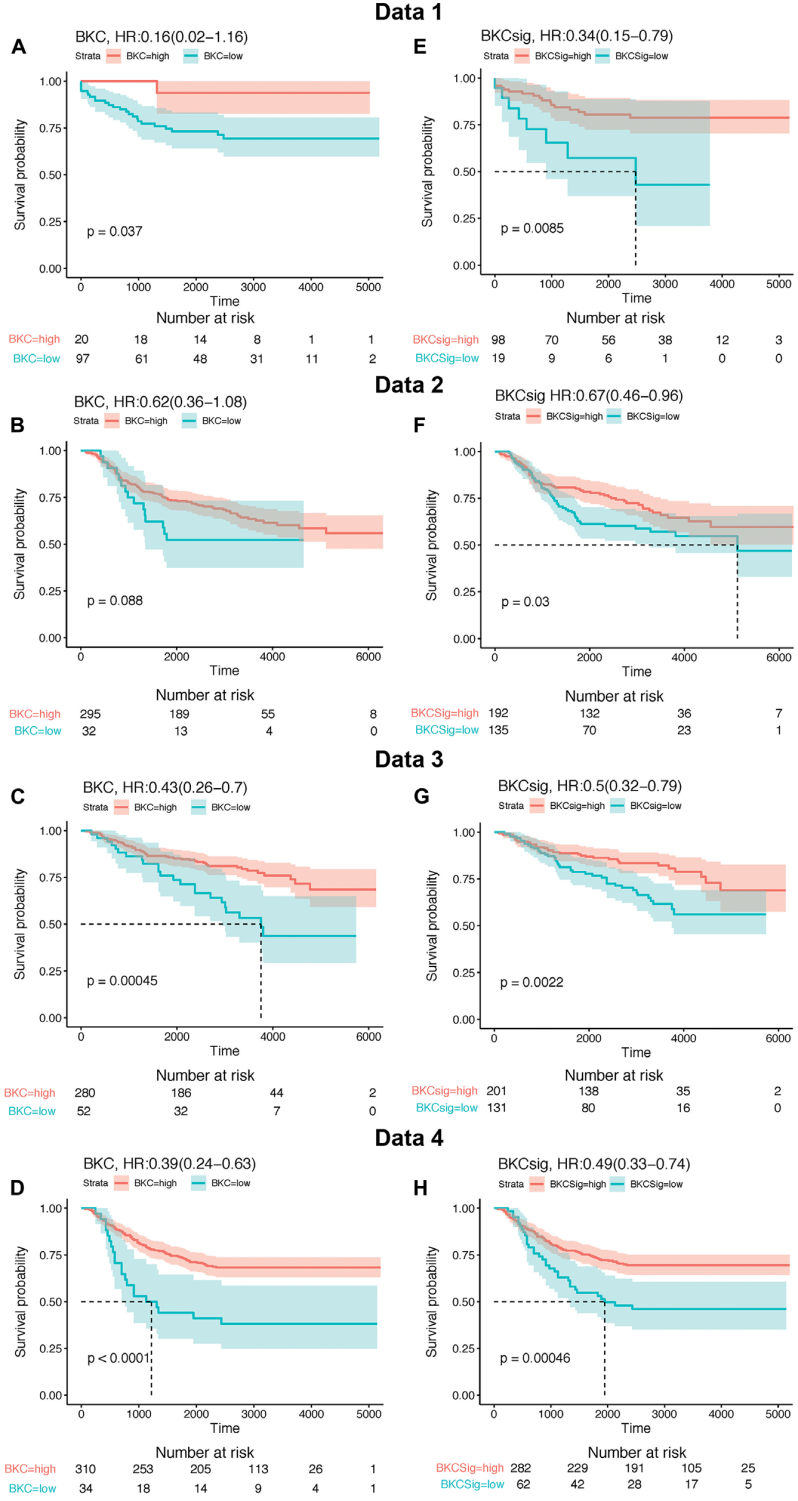
Kaplan–Meier (KM) survival analysis of BKC gene and BKCsig gene signature using four independent publicly available breast cancer relapse free survival patient cohorts named as Data 1, Data 2, Data 3, and Data 4. Maximally selected rank statistic was applied to define optimal H-score cut-off for Kaplan–Meier survival analysis of breast cancer free survival for low BKC and BKCsig gene signatures. (**A**–**D**), KM survival plot for BKC gene signature on Data 1, Data 2, Data 3, and Data 4 cohort, respectively. (**E**–**H**). KM survival plot for BKCsig on Data 1, Data 2, Data 3, and Data 4 cohort, respectively.

An analysis was performed to determine the ability of the BKC and BKCsig sets of genes to correlate with breast cancer patient survival. Four public databases (Data 1 [25], Data 2 [26], Data 3 [27], and Data 4 [28, 29]) were used to compute gene signature scores for both the BKC and BKCsig sets of genes (Figure 5). The BKC and BKCsig gene sets were both able to separate breast cancer patients into classifications of good versus poor survival in all four sets of patient data. For both the BKC (Figure 5A–5D) and BKCsig (Figure 5E–5H) datasets, the lower value of gene expression significantly correlates to poor survival in all four cohorts (Data 1–4). These results suggest that Cd^2+^- induced transformation of MCF-10A cells produces a reduced gene expression profile of keratinization associated with poor survival in breast cancer patients.

### Expression of basal genes in breast cancer cell lines

**Figure 6:**
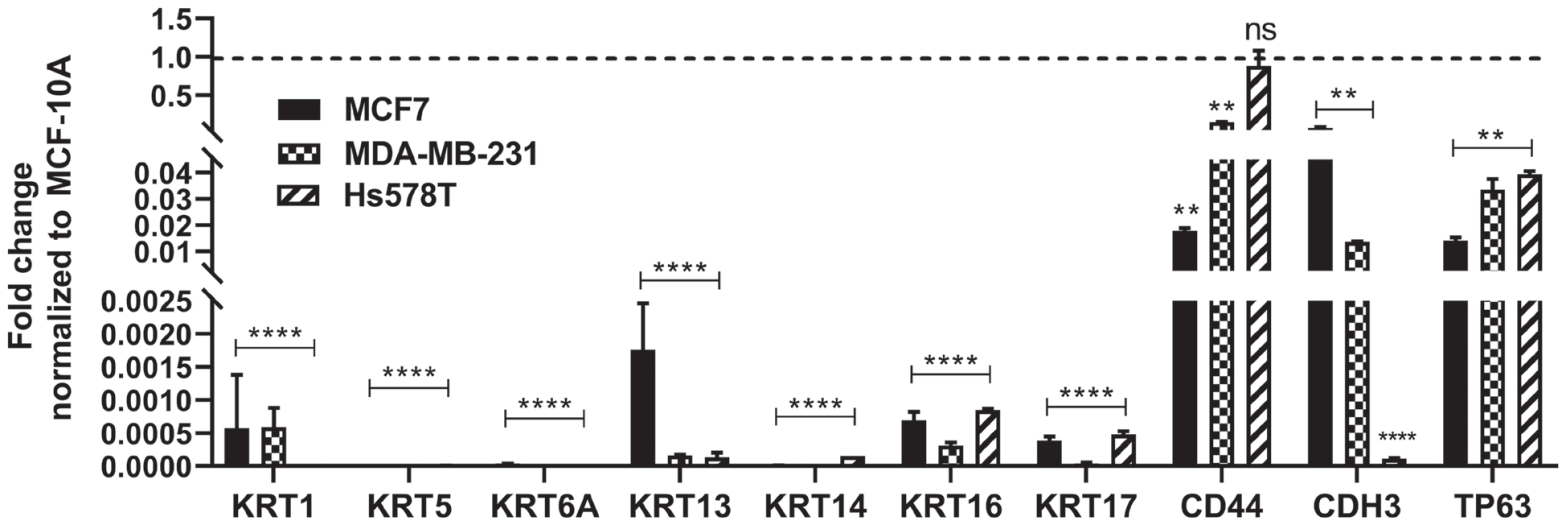
Expression of basal markers in breast cancer cell lines. Real time RT-PCR analysis of KRT1, KRT5, KRT6A, KRT13, KRT14, KRT16, KRT17, CD44, CDH3, and TP63 in the breast cancer cell lines, MCF7, MDA-MB-231, and Hs578T. The analysis was done in triplicates and is plotted as the mean ± SE. The expression level of each gene in the MCF7, MDA-MB-231, and Hs578T cells is normalized to β-actin and is plotted as fold-change relative to MCF-10A cells. The dotted line represents normalized value of gene expression in MCF-10A cells. ^****^; ^**^; ^*^Indicates significant difference in gene expression level compared to MCF-10A cells at *p*-value of ≤ 0.0001; ≤ 0.01; ≤ 0.05, respectively; ns represents change in expression is not significant.

The expression of KRT1, KRT5, KRT6A, KRT13, KRT14, KRT16, KRT17, CD44, CDH3, and TP63 genes were determined in the MCF7, Hs578T, and MDA-MB-231 cell lines (Figure 6). When compared to the parental MCF-10A cells (control), the expression of all the above basal genes were significantly reduced in all the cell lines, with the exception of CD44, which had similar gene expression levels in the Hs578t cell line when compared to the MCF-10A cells.

### Tumor formation by MCF-10ACd cells

**Figure 7:**
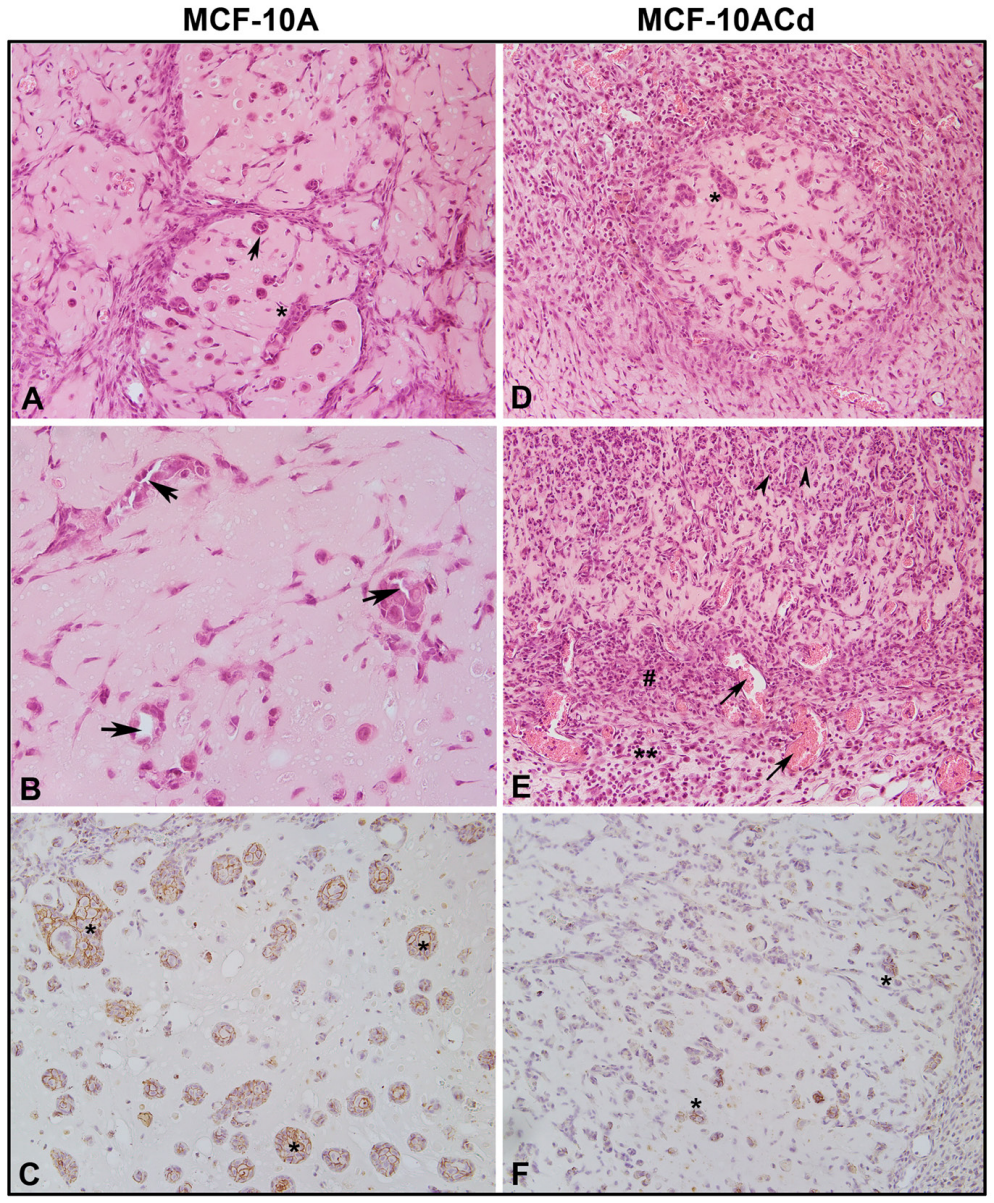
Histology and immunohistochemistry of nodules produced by MCF-10A and MCF-10ACd cells. (**A**). Hematoxylin and Eosin stained section of MCF-10A nodule. The arrow indicate epithelial ducts whereas the asterisk (^*^) indicates epithelial nests. The magnification of the image is at 200×. (**B**). Hematoxylin and Eosin stained section of MCF10-A nodule showing epithelial ducts with prominent central lumen indicated by arrows. The magnification of the image is at 400×. (**C**). Immunohistochemical staining for E-cadherin in MCF-10A nodule showing epithelial nests marked by ^*^. The magnification of the image is at 200×. (**D**). Hematoxylin and Eosin stained section of MCF-10ACd nodule. ^*^indicates epithelial nests. The magnification of the image is at 200×. (**E**). Hematoxylin and Eosin stained section of MCF-10ACd nodule showing necrosis and granulation areas. Arrow heads indicate necrotic cells. ^#^Indicates granulation tissue with newly formed blood vessels marked by arrows. ^**^Indicates inflammatory cells. The magnification of the image is at 200×. (**F**). Immnohistochemical staining for E-cadherin in MCF-10ACd cells.^*^Indicates staining of epithelial cells. The magnification of the image is at 200×.

Our previous studies show that MCF-10ACd cells readily form colonies in soft agar, but do not form tumors when injected subcutaneously into immune compromised mice [15]. This is in contrast to one other study where the authors show that Cd^2+^- transformed MCF-10A cells form tumors capable of metastasis when placed under the renal capsule of immune compromised mice [30]. To address this discrepancy, the laboratory took advantage of the ability of immortalized cells to form nodules when injected subcutaneously with matrigel into immune compromised mice. These nodules form 5–8 days post injection and start to regress after 8–12 days. The cellular morphology within these nodules is being used by this laboratory [31, 20], and others [32] to judge the ability of cells to form differentiated structures. Thus, the ability to form nodules with histologically differentiated structures was determined for the MCF-10 parent and the MCF-10ACd cells. Figure 7 shows the histology of the nodules formed by the parent and the transformed cells. The parent MCF-10A cells formed multiple nests of epithelial cells and occasional ducts with clear lumens consistent with differentiation of the cells (Figure 7A and 7B, respectively). The ducts were few in number, but there was no evidence of necrotic cells, cellular debris, or immune cell infiltration. Immuno-histochemical staining for E-cadherin confirmed the epithelial identity of the MCF-10A cells (Figure 7C) which formed cellular nests and ductal structures (Supplementary Figure 1). The nodules formed by the MCF-10ACd cells had few epithelial nests with areas of necrosis and granulation tissue with infiltration with a large number of inflammatory cells (Figure 7D and 7E and Supplementary Figures 2 and 3). There were no ductal structures formed by the MCF-10ACd cells. Immuno-histochemical staining for E-cadherin (Figure 7F) showed a few epithelial nests which were smaller in size when compared to the nests formed by the MCF-10A cells and no ductal structures (Supplementary Figure 4).

**Figure 8:**
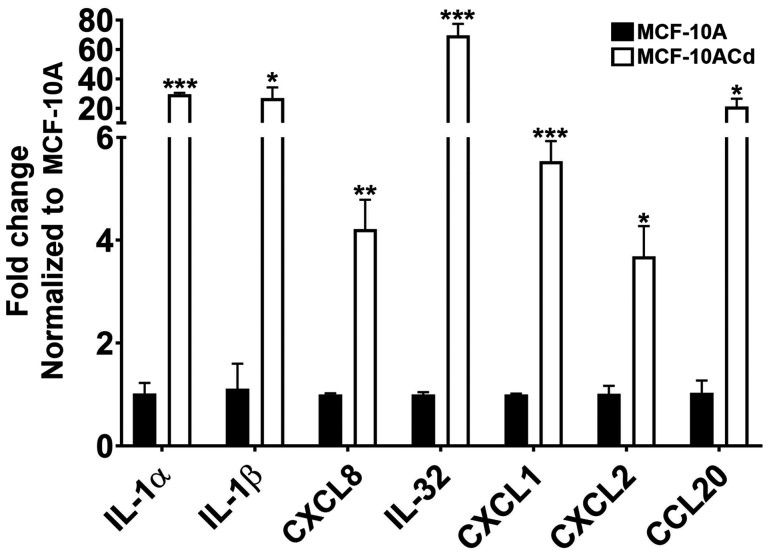
Gene expression analysis of inflammatory cytokines and chemokines in MCF-10A parent cells and MCF-10ACd. Real time RT-PCR analysis was performed to verify gene expression. Gene expression was normalized to β-actin and are plotted as fold-change relative to the MCF-10A parent cells. Triplicate measurements of gene expression was performed and are reported as mean ± SEM. Unpaired *t*-test was performed to determine statistical significance. ^***^; ^**^; ^*^Indicates significant difference in gene expression level compared to the MCF-10A parent cells at *p*-value of ≤ 0.001; ≤ 0.01; ≤ 0.05, respectively.

A preliminary analysis was performed to determine if MCF-10ACd cells expressed genes for immune-related mediators that were recruiting macrophages and neutrophils to the site of the nodule causing necrosis of the tumor cells. Supplementary Table 2 lists the chemical mediators with increased expression in the MCF-10ACd cells compared to the parent MCF-10A cells. The genes were confirmed using real-time RT-PCR and IL-1α, IL-1β, CXCL8, IL-32, CXCL1, CXCL2, and CCL20 were expressed at high levels in the MCF-10ACd cells when compared to the MCF-10A parent cells (Figure 8). The rest of the genes did not show a change in expression.

## DISCUSSION

There are limited cell culture models of the “normal” mammary epithelial cell for use in breast cancer research. As detailed in the introduction, the immortalized MCF-10A cell line is frequently used as a cell culture model of the “normal” breast epithelial cell [16, 17]. Another cell culture system used to model normal breast epithelium is the primary cultures of human mammary epithelial cells. A limitation of these models is that they have the gene expression pattern of basal epithelial cells. This is an important distinction since the majority of breast cancers are adenocarcinomas that arise from the duct-lobular unit of the breast [33]. However, a recent study profiling human breast epithelial cells using single cell RNA sequencing provides evidence that connects the basal lineage to the two different luminal branches [34]. Another important fact is that malignant transformation of these two cell culture models produce tumor transplants with squamous differentiation, instead of the expected adenocarcinomas [35, 18]. There has been one other study where MCF-10A cells transformed with Cd^2+^ [30] produce tumor transplants without any evidence of squamous differentiation. The tumor cells appear as undifferentiated epithelial cells that form lymph node metastases. Based on these findings, our lab performed the current study to determine if transformation of MCF-10A cells with Cd^2+^ effects the expression of basal genes in the MCF-10A cells and elucidates the role of Cd^2+^ in the development and progression of breast cancer.

A very simplistic view of the current data would be that MCF-10A cells model some stage of basal cell differentiation and that Cd^2+^ transformation can change the stage by decreasing the expression of genes associated with a basal genotype. Evidence that this decrease in expression may be an early step in carcinogenesis is suggested by the finding that the basal genes assessed in the present study also had very low expression in the three commonly used breast cancer cell lines; MDA-MB-231, MCF-7 and Hs578t. All three of these cell lines have decrease expression of basal genes, even though the MDA-MB-231 and Hs578t cells form aggressive tumors in immune compromised mice. Since each of the cell lines vary significantly in their differentiated states, our finding suggests that Cd^2+^ may play an early role in the promotion of breast cancer by decreasing the expression of basal genes.

The finding that Cd^2+^ transformation decreases basal gene expression would be enhanced if these gene signatures had an impact on patient outcome. An examination of existing publically available breast cancer patient databases showed that the gene signature associated with basal differentiation and keratinization identified using MCF-10ACd cells had an impact on patient survival. The impact on patient survival was significant for both the 29 gene BKC gene signature based on pathway analysis and the BKCsig gene signature based on a subset of BKC genes that were significant when analyzed independently for patient survival. The correlation of these basal genes with patient survival strengthens the possibility that exposure to Cd^2+^ could play a role in the development of breast cancer. In almost all instances, the expression of the BKC genes in the MCF-10ACd cells decreased, suggesting the transformed cells were “less basal” than the MCF-10A parent cells. The expression of the basal genes were even lower in the MCF-7, Hs578t, and MDA-MB-231 cell lines. In addition, the expression of both the BKC and BKCsig signatures positively correlated only with basal-like breast cancers. There was no association of these gene signatures with the ER status or the histological grade of the tumor. The only association noted was for the early response (proliferation) and lipid modules. Overall, the decrease in expression of basal genes in Cd^2+^- transformed MCF-10A cells identify a set of genes associated with the outcome of breast cancer patients.

Cadmium can accumulate in the human breast due to the presence of the metallothionein (MTs), a family of low molecular weight proteins that bind seven molecules of zinc (Zn^2+^) and are involved in the regulation of Zn^2+^ concentrations within the cell. They can also bind seven molecules of Cd^2+^ allowing the pollutant to concentrate in cells expressing the MTs and organ concentrations of Cd^2+^ increase over the lifespan [36]. In breast tissue, only the myoepithelial cells express high levels of MT proteins [37–39], whereas both the myoepithelial as well as the ductal epithelial cells express high levels of MT mRNA [39]. This affords the breast epithelial cell the potential to translate the MT mRNA to protein under the appropriate conditions. In breast cancer, there is overexpression of MT protein in both ductal carcinoma *in situ* and invasive ductal breast cancer [40–43] and this overexpression in ductal breast carcinoma correlates to poor histological type and grade [43].

Several mechanisms are proposed by which MT overexpression and/or its binding to Cd^2+^ can elicit cellular dysregulation that would promote cancer development and progression. These mechanisms involve the ability of MT to provide Zn^2+^ to the zinc fingers of numerous transcription factors and the p53 protein. One view is that overexpression of MT could produce an excess of apoMT and sequester Zn^2+^ from being available to zinc-requiring proteins. Earlier studies demonstrated this possibility by showing that the microinjection of apoMT into living cells was capable of removing Zn^2+^ from the zinc finger DNA binding proteins, Sp-1, and transcription factor IIIA [44–47]. A related hypothesis is that MT saturated wholly or partially with Cd^2+^, could substitute Cd^2+^ in place of Zn^2+^ in zinc requiring proteins and alter their regulatory function. MT’s have been shown to mediate the activity of p53 by removal of Zn^2+^, leading to changes in its spatial structure and loss of function, similar to p53 mutations. Meplan and coworkers [48, 49] demonstrated that MT overexpression exerted a potent inhibitory effect on transcriptional activity of p53, consistent with a metal chelating or substitution effect on p53. Two additional early reports summarized the possible role of MT and its effect on p53 in breast cancer cells [50, 51]. These mechanisms while plausible, have not been proven or dismissed and remain the subject of speculation. However, they could explain, based on Zn^2+^ homeostasis, the variety of cellular processes that are associated with the expression of the MT’s. As recently reviewed, these include drug resistance in many cancers; regulation of tumor cell growth through various pathways; tumor metastasis; tumor and tissue angiogenesis; cellular differentiation; and, immunomodulation [52].

The present study also identified the increased expression of three cytokines (IL-1α, IL-1β and IL-32) and four chemokines (CXCL1, CXCL2, CXCL8, also known as IL-8 and CCL20) in the MCF-10ACd cells when compared to the parent MCF-10A cells. This increased expression of immune mediators could explain the lack of survivability of the MCF-10ACd cells when injected subcutaneously into nude mice. The parent MCF-10A cells when injected subcutaneously with matrigel differentiated and formed duct like structures whereas the Cd^2+^-transformed cells formed few epithelial nests with necrotic areas and granulation tissue present in the nodule. The cytokines and chemokines produced by the transformed cells are pro-inflammatory and are chemotactic for neutrophils and monocytes/macrophages and these cells could contribute to the necrosis of the transformed cells. The effect of these cytokines and chemokines could explain the lack of tumor formation by the MCF-10ACd cells [15]. Another group of investigators were successfully able to transform MCF-10A cells with Cd^2+^ and these transformed cells were tumorigenic [30]. However, the transformation was done with 5 μM Cd^2+^, where our transformation was done with the environmentally relevant dose of 1 μM Cd^2+.^ Additionally, we terminated exposure to Cd^2+^ once the cells formed colonies in soft agar whereas in the study performed by Benbrahim-Tallaa and coworkers [30], exposure was extended for a considerable length of time. At present, we do not know if the concentration and duration of exposure would influence the expression of various genes in the MCF-10A cells as well as other models of breast cancer.

In conclusion, our study shows that exposure of the MCF-10A cells long-term to environmentally relevant doses of Cd^2+^ decreases the expression of genes associated with the basal sub-type of breast cancer. In addition, the increased expression of the BKC and the BKCsig gene signatures in human breast cancer patients positively correlates with increased survival and the absence of this signature is associated with poor survival.

## MATERIALS AND METHODS

### Cell culture

The MCF-10A cell line was obtained from the American Type Culture Collection and grown in a 1:1 mixture of Ham’s F-12 medium and Dulbecco’s Modified Eagle Medium (DMEM) supplemented with 5% (v/v) fetal calf serum, 10 μg/ml insulin, 0.5 μg/ml hydrocortisone, 20 ng/ml epidermal growth factor, and 0.1 μg/ml cholera toxin. MCF-10A cells transformed previously were used in this study and the methodology for transformation with Cd^2+^ has been discussed in our previous publication [15]. The MCF7, Hs578T, and MDA-MB-231 breast cancer cell-lines obtained from the American Type Culture Collection were grown in DMEM supplemented with 10% (v/v) fetal calf serum, as described previously [53].

### Global gene expression

RNA was purified from triplicate cultures of the MCF-10A and MCF-10A cell line transformed by exposure to Cd^2+^ by the RNeasy Mini kit (Qiagen). Purity and concentration of RNA samples were determined from OD_260/280_ readings using a dual beam UV spectrophotometer. For each cell line, aliquots of triplicate samples of RNA were mixed in equal amounts (1:1:1) before submission for array analysis and they represented one sample for array hybridization. Global gene expression analysis was performed by Genome Explorations Inc. (Memphis, TN, USA). The RNA integrity was determined by capillary electrophoresis using the RNA 6000 Nano Lab-on-a-Chip kit and the Bioanalyzer 2100 (Agilent Technologies) as per the manufacturer’s instructions. For cRNA synthesis and labeling, the RNA was processed and labeled according to standard RTIVT methods as described previously [54]. The fragmented cRNA was hybridized for 16 h at 45°C to GeneChip Human Genome U133 Plus 2.0 arrays (Affymetrix, Santa Clara, CA, USA). The arrays were stained with phycoerythrein-conjugated streptavidin (Invitrogen, Carlsbad, CA, USA) and the fluorescence intensities were determined using a GCS 3000 7G high-resolution confocal laser scanner (Affymetrix). The scanned images were analyzed using programs resident in Gene Chip Operating System v1.4 (GCOS; Affymetrix). Quality control metrics for cRNA integrity, sample loading, and variations in staining were determined after background correction and signal summarization by MAS 5.0 statistical algorithms resident in GCOS and standardization of each array by global scaling the average of the fluorescent intensities of all genes on an array to a constant target intensity (TGT) of 250. Differentially expressed genes (DEGs) were identified using empirical Bayes (EBayes) method and the *p* values were adjusted using false discovery rate [55, 56]. The DEGs were further evaluated using the Reactome Pathway Knowledge Base and the DAVID Bioinformatics resources [54, 57]. The results were validated using the publicly available functional genomics data repository Gene Expression Omnibus GEO (link is https://www.ncbi.nlm.nih.gov/geo/). Statistical analysis was performed using Graphpad PRISM and R/Bioconductor version 3.5.1.

### Survival analysis for a single gene

The Kaplan–Meier plotter (KMPlotter, http://kmplot.com) was used to perform breast cancer survival analysis for each of the single gene using the gene expression data derived from the Affymetrix microarrays only and using relapse free survival (rfs) as an outcome. The clinical characteristics are available in the above mentioned website to classify the patients based on estrogen receptor (ER status). The patient samples were divided into two groups to assess the good versus poor prognostic value based on the median expression cutoff of the proposed gene. The hazard ratio (HR) with 95% confidence intervals (CI) and log rank *P* value were calculated to examine the prediction ability. The analysis was performed in three steps: 1) All available samples (*n* = 3779); 2) Only ER positive samples (*n* = 2565); and 3) Only ER negative samples (*n* = 1214).

### Survival analysis of gene signatures

To test the combined detection power, two gene signatures were created; 1. Using all 29 genes and this was named as Basal Keratinization Cluster (BKC) signature, and 2. Using only the significant 14 (survival *p* value < 0.01) genes and this was named as BKCsig signature. Kaplan–Meier survival plots were generated using four different publicly available patient cohorts, and the hazard ratio HR with 95% confidence intervals (CI) and logrank-*P* value were calculated using R package survival. Each database was normalized and pre-processed before adding to the data pool. To classify the patient into high vs low signature score, optimal cut-off points for H-score were determined as previously described [58, 59] (Supplementary Figure 5). For the Signature Score Calculation, raw data was retrieved from four public databases: (Data 1, *n* = 118 [25],); (Data 2, *n* = 295 [26],); (Data 3, *n* = 255 [27],); and, (Data 4, *n* = 344 [28, 29], which were obtained from GEO or ArrayExpress and normalized using R Affy package. To compute gene signature scores for both our gene signatures BKC and BKCsig, a previously described method [60] was used that is based upon weighted average. Each module score was scaled within the study so that the 2.5% and 97.5% quantiles equaled +1 and −1, respectively. The entire analysis was performed using R/Bioconductor (www.r-project.org).

### Breast cancer subtype analysis

Our previous studies demonstrated that the single genes and gene signatures often represent the same biological processes [60–62] are more significantly associated with outcome only in specific sub-groups of breast cancer (i.e., Luminal A, Luminal B, HER2+, Basal, and Normal like), and validation of such gene signatures needs to be performed with independent large cohorts [63]. Therefore, we have used: 1) TCGA dataset [64] with 1102 breast cancer (BC) samples to find-out the breast cancer subgroup association with each independent gene; 2) GOBO [65] tool to perform sample prediction analysis to determine the influence of specific subtype on both gene signatures with a breast cancer data set containing 1881 samples. For sub-classification of the TCGA BC population, PAM50 subtype was used as described in our previous publication [60]. The GOBO application used PAM50 and the Hu et al. [66] gene expression subtype classification approach to classify the subtype population of BC patients. Rather than using counts, FPKM (fragments per kilobase of exon model per million reads mapped) a normalized estimation of gene expression based on RNA-seq data value was used as the measure of gene-expression.

### RNA isolation and qPCR

Total RNA was isolated using Tri Reagent (Molecular Research Center, Inc., Cincinnati, OH, USA). The measurement of mRNA expression of selected genes was assessed using RT-PCR and commercially available primers (Bio-Rad Laboratories, Hercules, CA, USA). For analysis, 0.1 μg of total RNA was subjected to complimentary DNA synthesis using the iScript cDNA synthesis kit (Bio-Rad Laboratories, Hercules, CA, USA) in a total volume of 20 μl. Real-time PCR was performed utilizing the SYBR Green kit (Bio-Rad Laboratories) with 2 μl of cDNA, 0.2 μM primers in a total volume of 20 μl in an iCycler iQ real-time detection system [19].

### Animal studies

Athymic Nude-Foxn1^nu^ mice from ENVIGO were purchased for use in these studies. The mice were housed at 22°C under a 12-hour light/dark cycle. Food and water was available *ad libitum*. Confluent cultures of MCF-10A parent and the Cd^2+^-transformed cells were trypsinized and cell pellets were re-suspended in ice-cold phosphate buffered saline PBS and mixed with an equal volume of ice-cold Corning matrigel (Corning, NY, USA). 1 × 10^6^ cells in a 0.2 ml total volume was injected subcutaneously in the dorsal thoracic midline of mice using a 0.2 cc syringe. The matrigel nodules were harvested seven days post injection. The study adhered to all recommendations dictated in the Guide for the Care and Use of Laboratory Animals of the NIH. The specific protocol was approved by the University of North Dakota Animal Care Committee (IACUC#1911-1C).

## SUPPLEMENTARY MATERIALS



## Abbreviations

As^3+^: arsenite
BKC: Basal Keratinization Cluster
BKCsig: Basal Keratinization Cluster Sig
Cd^2+^: cadmium
BC: breast cancer
CI: confidence intervals
DEGs: Differentially expressed genes
DMEM: Dulbecco’s Modified Eagle Medium
ER: estrogen receptor
EBayes: Empirical Bayes
FPKM: fragments per kilobase of exon model per million reads mapped
GCOS: Gene Chip Operating System v1.4
HR: hazard ratio
KMPlotter: Kaplan–Meier plotter
MT: metallothionein
MIBC: muscle-invasive bladder cancer
PR: progesterone receptor
Zn^2+^: zinc
