# Supplementary material for: Meta-analysis of gene expression profiling reveals novel basal gene signatures in MCF-10A cells transformed with cadmium
Source: Oncotarget. 2020 Sep 29;11(39):3601–17. doi: 10.18632/oncotarget.27734 (PMC7533076; doi:10.18632/oncotarget.27734)
Supplement: Supplementary file 1 [file oncotarget-11-3601-s001.pdf]

Meta-analysis of gene expression profiling reveals novel basal gene signatures in MCF-10A cells transformed with cadmium

SUPPLEMENTARY MATERIALS

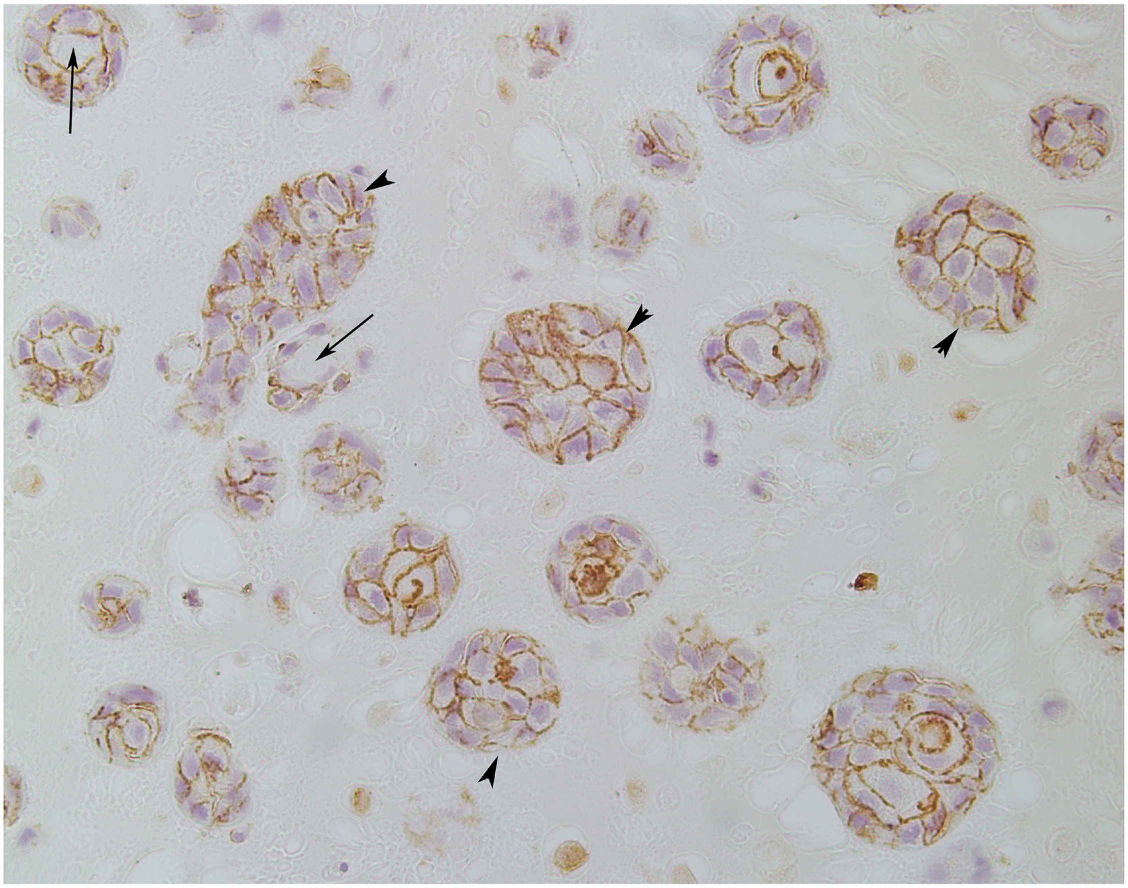

Supplementary Figure 1: Immunohistochemical staining for E-Cadherin in MCF-10A nodule. The epithelial nests are marked by arrowheads, whereas the epithelial ducts with a central lumen are marked by arrows. The magnification of the image is at 400×.

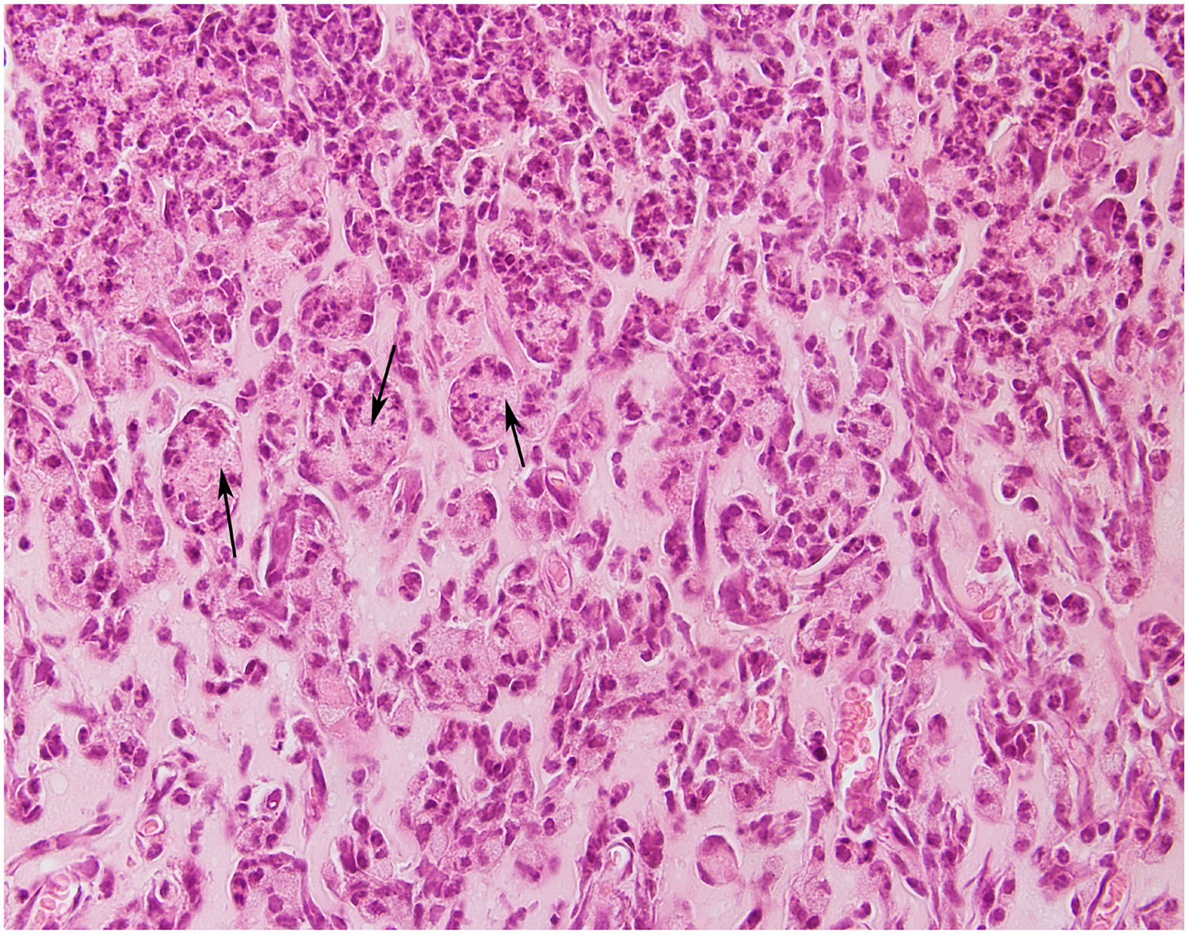

Supplementary Figure 2: Hematoxylin and Eosin stained section of MCF-10ACd nodule showing necrotic area. The arrows indicate necrotic areas within the epithelial nests with loss of nuclear material. The magnification of the image is at 400×.

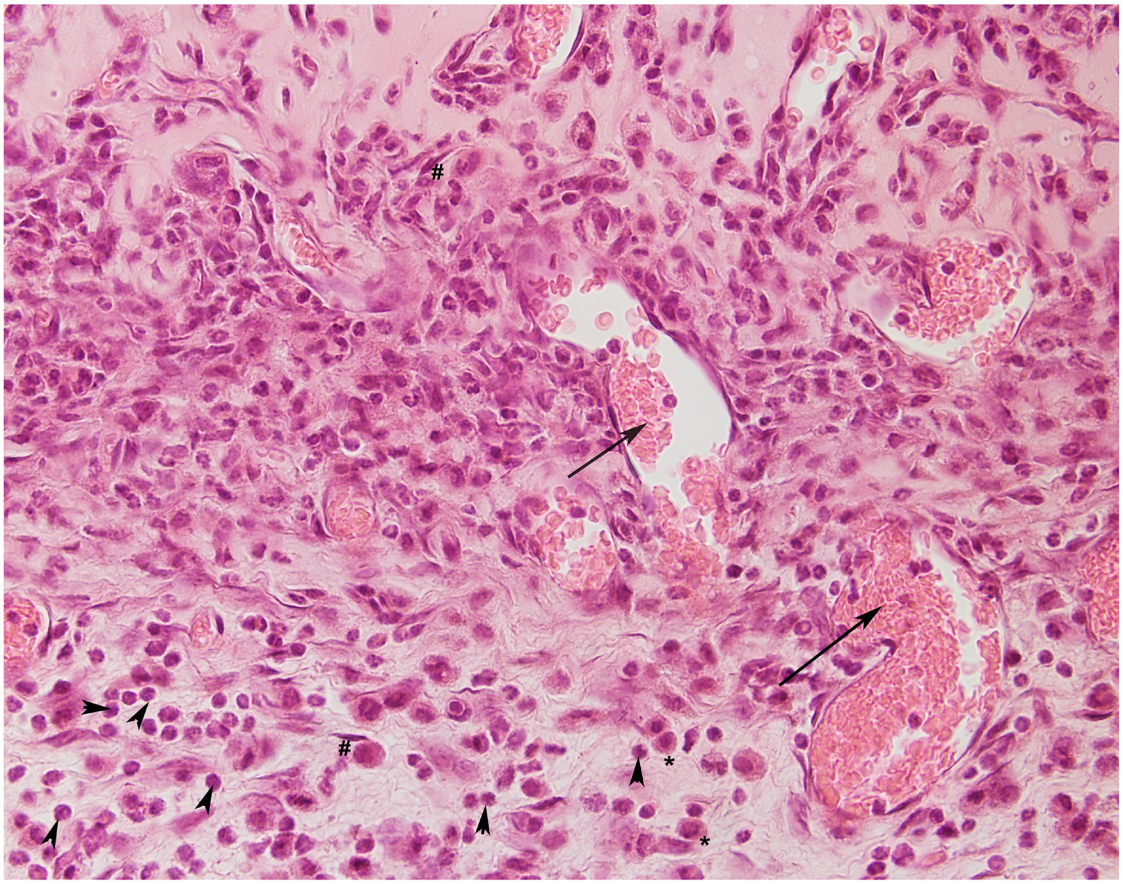

Supplementary Figure 3: Hematoxylin and Eosin stained section of MCF-10ACd nodule showing granulation tissue. The newly formed blood vessels are indicated by arrows. The lymphocytes are marked by arrowheads whereas the macrophages are indicated by asterisks*. The fibroblasts are marked by the # sign. The magnification of the image is at 400×.

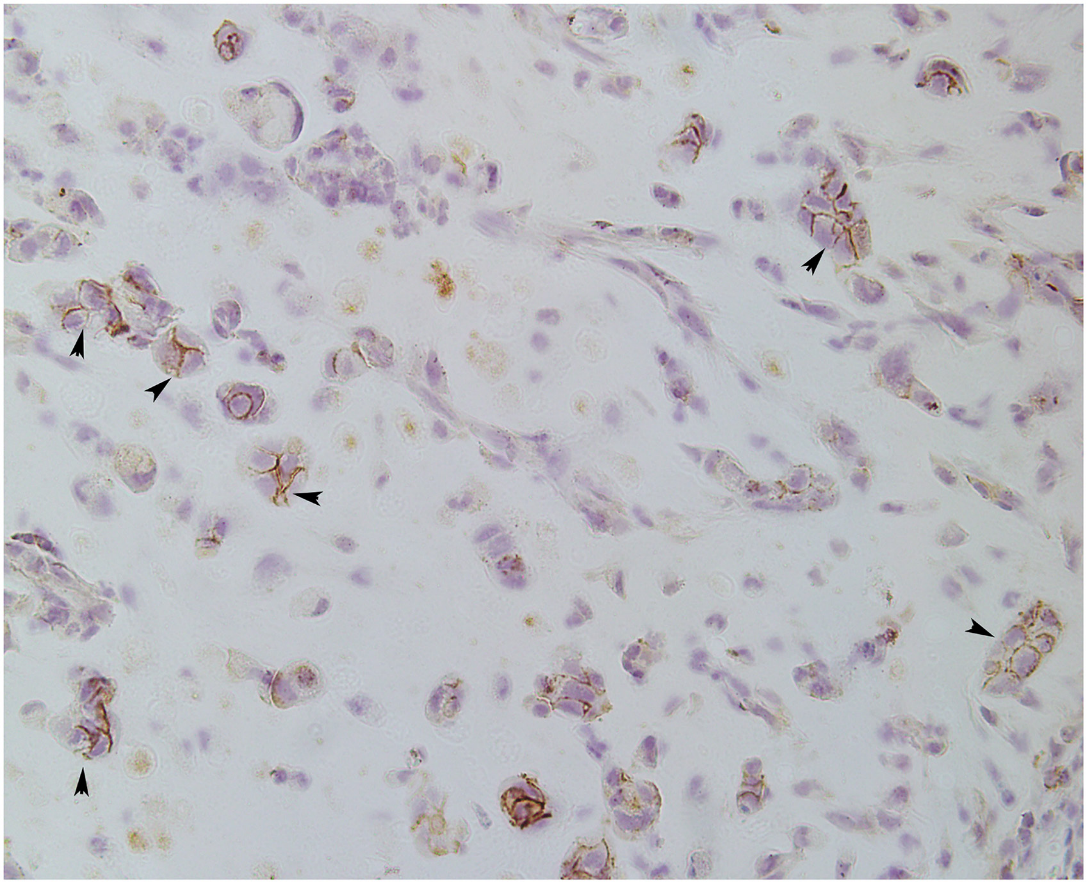

Supplementary Figure 4: Immunohistochemical staining for E-Cadherin in MCF-10ACd nodule. The epithelial nests are marked by arrowheads. The magnification of the image is at 400×.

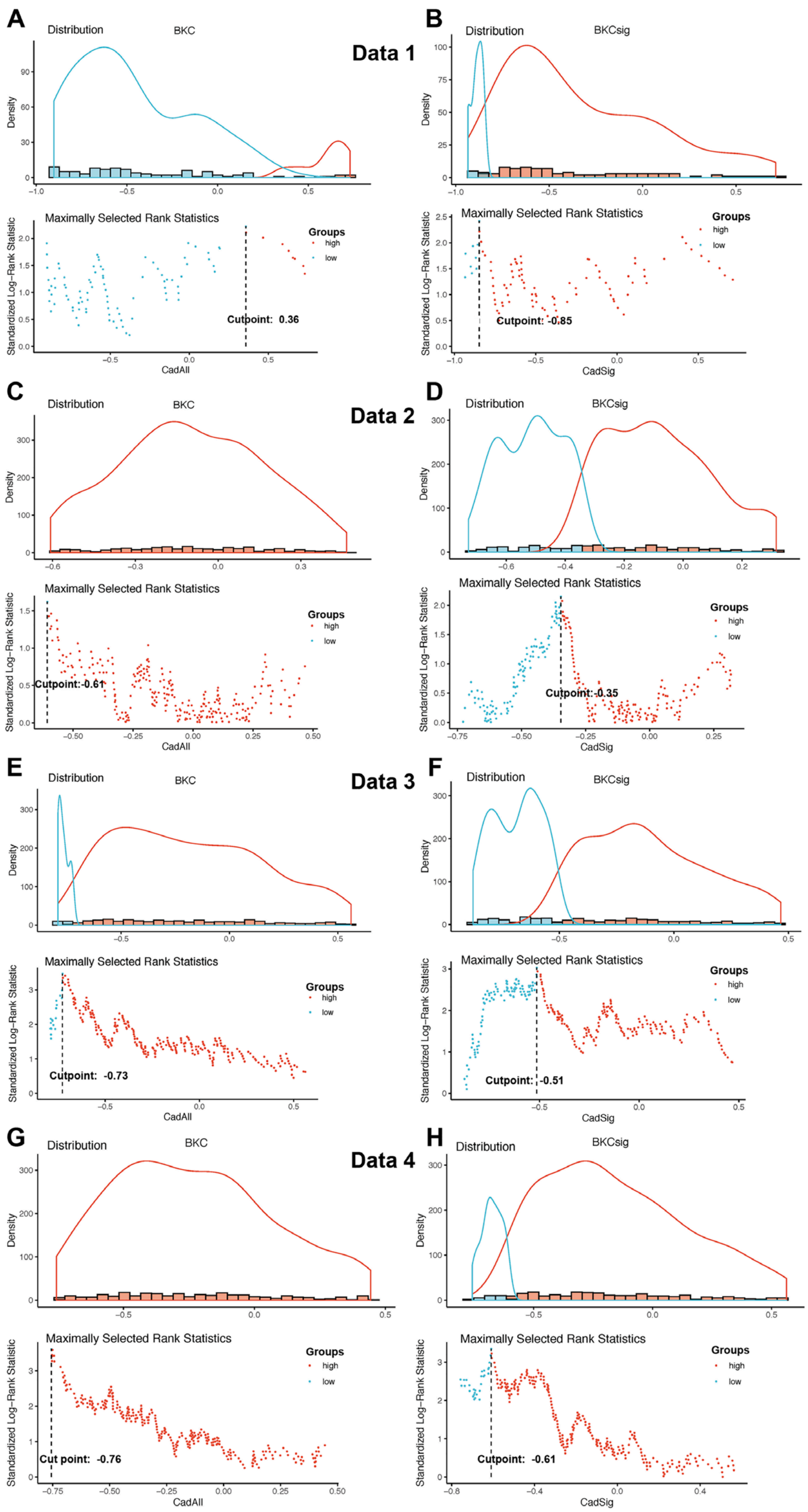

Supplementary Figure 5: Distribution of data based on optimal H-score cut-off approach for Kaplan–Meier survival analysis. The solid lines and histogram present data for samples with levels higher (red) or (lower). The broken line represents data for samples divided into two groups (higher; red or lower; blue) based on the optimal cut off algorithm. (**A**–**D**), distribution of data based on optimal H-score cut-off for BKC gene signature on Data 1, Data 2, Data 3, and Data 4 cohort respectively. (**E**–**H**). Distribution of data based on optimal H-score cut-off for BKCsig on Data1, Data 2, Data 3, and Data 4 cohort, respectively.

Supplementary Table 1: Cornified envelope gene signature of MCF-10ACd cells

Gene Symbol	Gene Description	Fold change compared to MCF-10A
PI3	Peptidase inhibitor 3, Elastase specific inhibitor	−4.2
KRT6A	Keratin involved in squamous differentiation	−1.3
DSP	Desmoplakin, anchors intermediate filaments to desmosomal plaques	−1.7
TGM1	Transglutaminase 1, catalyzes the cross linking of epidermal proteins	−6.3
SPINK6	Serine protease inhibitor kazal-type 6, involved in keratinization of skin	−3.7
KRT6B	Keratin involved in squamous differentiation	−1.6
SPRR1A	Small proline rich protein 1A, cross-linked envelop protein of keratinocytes	−7.4
SPRR1B	Small proline rich protein 1B, cross-linked envelop protein of keratinocytes	−7.0
KRT14	Keratin, participates with KRT5 to form epithelial cytoskeletal structure	−7.3
CAPN1	Calpain 1, catalyzes limited proteolysis of substrates involved in cytoskeletal remodeling	−1.2
KRT16	Keratin, regulator of innate immunity in response to skin barrier breach	−8.8
JUP	Junction plakoglobin, catenin family member that forms distinct complexes with cadherins	−1.3
KRT10	Keratin, component of the cytoskeleton of epithelial cells	−2.1
KRT13	Keratin, expressed in the suprabasal layers of non-cornified stratified epithelium	−5.3
KRT1	Keratin, specifically expressed in the spinous and granular layers of the epidermis	−1.5
SPRR3	Small proline rich protein 3, cross-linked envelope protein, component of the cornified cell envelope	−4.2
KRT5	Keratin, specifically expressed in the basal layer of the epidermis	−.2.7
PKP3	Plakophilin, links cadherins to intermediate filaments in the cytoskeleton	−1.0
DSG3	Desmoglein3, interacts with plaque proteins and intermediate filaments in cell-cell adhesion	−2.2
SPTAN1	Spectrin 1, functions as a scaffold protein that stabilizes the plasma membrane	−1.5
CSTA	Cystatin A, functions as a cysteine protease inhibitor in the formation of the cornified envelope	−1.5
DSC1	Desmocolin-1, adhesive protein of the desmosome cell-cell junction	−1.7
DSC2	Desmocolin-2, adhesive protein of the desmosome cell-cell junction	−1.9
DSC3	Desmocolin-3, adhesive protein of the desmosome cell-cell junction	−2.2
KLK5	Kallikrein 5, secreted and may be involved in desquamation of epidermis	−2.7
PERP	Effector in the TP53-dependent apoptotic pathway, participates in stratified epithelial integrity	−1.5
PPL	Periplakin, component of desmosomes and of the cornified envelope in keratinocytes	−2.9
KRT6C	Keratin involved in squamous differentiation	−1.0
KRT17	Keratin involved in squamous differentiation	−1.0

Supplementary Table 2: Cytokines and chemokines produced by the MCF-10ACd cells

Gene Symbol	Gene Description	Fold change compared to MCF-10A Cells
CXCL8	Chemokine (C-C motif) ligand 8	6.1
CCL20	Chemokine (C-C motif) ligand 20	6.3
CXCL1	Chemokine (C-X-C motif) ligand 1 (melanoma growth stimulating activity, alpha)	3.6
IL-1α	Interleukin 1, alpha	3.5
CXCL3	Chemokine (C-X-C motif) ligand 3	3
IL-32	Interleukin 32	3
CXCL2	Chemokine (C-X-C motif) ligand 2	3
IL-1β	Interleukin 1, beta	2.6
TNFAIP3	Tumor necrosis factor, alpha-induced protein 3	1.9
TGFB1I1	Transforming growth factor beta 1 induced transcript 1	1.6
IL-15	Interleukin 15	1.3
